# Limits and Trade-Offs of Topological Network Robustness

**DOI:** 10.1371/journal.pone.0108215

**Published:** 2014-09-24

**Authors:** Christopher Priester, Sebastian Schmitt, Tiago P. Peixoto

**Affiliations:** 1 Institut für Festkörperphysik, Technische Universität Darmstadt, Darmstadt, Germany; 2 Honda Research Institute Europe GmbH, Offenbach am Main, Germany; 3 Institut für Theoretische Physik, Universität Bremen, Bremen, Germany; Universidad de Zarazoga, Spain

## Abstract

We investigate the trade-off between the robustness against random and targeted removal of nodes from a network. To this end we utilize the stochastic block model to study ensembles of infinitely large networks with arbitrary large-scale structures. We present results from numerical two-objective optimization simulations for networks with various fixed mean degree and number of blocks. The results provide strong evidence that three different blocks are sufficient to realize the best trade-off between the two measures of robustness, i.e. to obtain the complete front of Pareto-optimal networks. For all values of the mean degree, a characteristic three block structure emerges over large parts of the Pareto-optimal front. This structure can be often characterized as a core-periphery structure, composed of a group of core nodes with high degree connected among themselves and to a periphery of low-degree nodes, in addition to a third group of nodes which is disconnected from the periphery, and weakly connected to the core. Only at both extremes of the Pareto-optimal front, corresponding to maximal robustness against random and targeted node removal, a two-block core-periphery structure or a one-block fully random network are found, respectively.

## Introduction

The theoretical investigation of complex networks has proven to be a valuable tool for the study of many real-world systems [1]–[6]. One important aspect is how the topological properties of networks are linked to their function and robustness [7], [8]. Robustness is defined as the correct functioning in the presence of disturbances, and it is a desired property of many empirical network systems. The robustness of networks to topological disturbances is a very active field of research [8]–[11], since it is often assumed that it is a necessary ingredient for higher-order forms of robustness associated with specific network dynamics [12]–[16].

One popular way to address topological robustness is by removing nodes from a given network and then analyzing how connected the network remains as function of the number of nodes removed [7], [17], [18]. In this way, the problem of robustness is mapped to the classical phenomenon of percolation, and the formation of a giant component in the remaining network after the node removals.

Recent studies focused on the optimization of the topological robustness of networks, when a given set of constraints are imposed [11], [19]–[25]. Most recent works have focused on optimization according to different robustness criteria, such as targeted attacks [11], [25], [26] and random failure [25], [26]. However, most real systems are subject to simultaneous types of perturbations, which individually require different, and thus competing strategies to mitigate failure. In order to properly access the inherent trade-offs in such situations, one needs to combine multiple robustness criteria. A standard technique is to chose a weighted sum of the relevant criteria as the objective function to be minimized or maximized. However, such an approach can be ineffective if the goal is to map all possible trade-off values between these objectives. In addition, it also bears the difficulty to define properly scaled objective functions for each criterion, such that a weighted sum really reflects the relative importance the multiple criteria.

In order to avoid such issues we use a multi-objective optimization approach [27]–[30], where a complete set of Pareto-optimal solutions is directly obtained. The two objectives we focus on are the topological robustness of networks against random and targeted removal of nodes. These two types of robustness are known to be in a trade-off relation, where increasing the robustness with respect to one type of removal is likely to decrease the other [18], [25], [26]. In particular, it has been recently shown that in absence of any constraints other than a fixed average degree, the optimization of robustness against random failure leads to a core-periphery structure, where most nodes are connected to a core group, possessing a high average degree, which is also internally connected [25]. Although being maximally robust against random failure, this core-periphery topology is minimally robust against targeted attacks, since the removal of the few core node immediately leads to the vanishing of the giant component. This robustness-fragility duality is a common feature of real networks with heterogeneous structure; a famous example of which is the Internet [31].

In order to investigate this multi-objective optimization scenario, we follow Ref. [25] and focus on large-scale topological features, as parametrized by a stochastic block model [32], [33]. This parametrization allows for arbitrary large-scale mixing patterns, such as assortativity, dissortativity, community structure, core-peripheries, etc., as well as arbitrary local degree distributions. This model is also convenient, since it allows the exact computation of the percolation properties of the system in the limit of large networks [25], [34].

By analyzing the Pareto-optimal fronts according to the two robustness criteria, we observe that a minimal number of three blocks is sufficient to obtain the optimal fronts, and that in most cases the best trade-off is realized by a hybrid structure composed of a core-periphery and a third “secluded” group, which is strongly connected internally and marginally connected to the core nodes. The two-block core-periphery of Ref. [25] and the fully random network are recovered at the two extremes of the Pareto fronts, for maximum robustness against random and targeted node removal, respectively.

This paper is organized as follows. In Sec. 1, we define the stochastic block model and in Sec. 2 our robustness criteria. In Sec. 3, the evolutionary multi-objective optimization algorithm is described briefly. Sec. 3 presents the results of the optimization for several parameter choices, including the Pareto-optimal fronts and the resulting topologies. In Sec. 5.3, we finalize with an overall discussion.

## Materials and Methods

### 1 Stochastic block model

The *stochastic block model* defines an ensemble of random networks, in which nodes belong to different groups (also called “blocks”), and the probability of an edge existing between nodes is a function of the block membership of each node. Each block holds a fraction 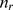 of the 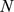 nodes of the whole network, where 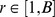 enumerates these blocks and 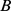 is the total number of blocks, such that 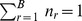. Following Ref. [33], each of the 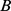 blocks is characterized by an independent degree distribution 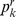, which specifies the fraction of nodes with degree 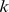 in block 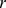.

The connections between the blocks are described with a matrix 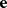, where the elements 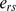 specify the number of half-edges per node in block 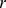 connecting to nodes in block 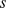. For simplicity of notation, the diagonal elements 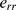 encode twice the number of edges per node within block 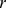.

In the framework of the stochastic block model, the network structure becomes locally tree-like when the number of nodes 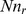 inside each block is sufficiently large. Since the probability of an edge existing between any two nodes of groups 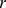 and 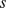 scales as is 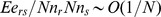, with 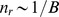 and 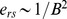, the probability of an edge existing between any two chosen neighbours will become vanishingly small as 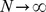. Therefore, since local substructures such as triangles are not generated by the model, predictions based on block model calculations can only be accurate for (large) locally tree-like networks without these local substructures. However, global and meso-scale properties such as community structure [33], assortativity [35], bipartite, core-periphery structures [25], or any other arbitrary mixing pattern are well captured.

Each block of the network can in principle have an arbitrary degree distribution 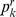. However, in this work we restrict the degree distribution of each block to be a modified Poisson degree distribution,
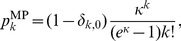
(1)where 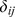 is the Kronecker delta function. Thereby nodes with zero degree (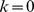) are explicitly excluded, since they can never belong to the giant component. In contrast to a regular Poisson distribution 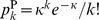, where the mean degree is directly given by 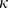, the mean degree of the modified Poisson distribution is given by 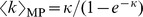, which always is greater than 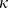. In particular, the mean degree cannot be less than one, 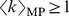.

It is important to note that although the use of the modified Poisson distribution as displayed in Eq. (1) may seem like a strong imposition on the network structure, in reality it is not. A large variety of nearly-arbitrary degree distributions of the complete network can be obtained by composing many blocks with different sizes and average degrees.

**Figure 1:**
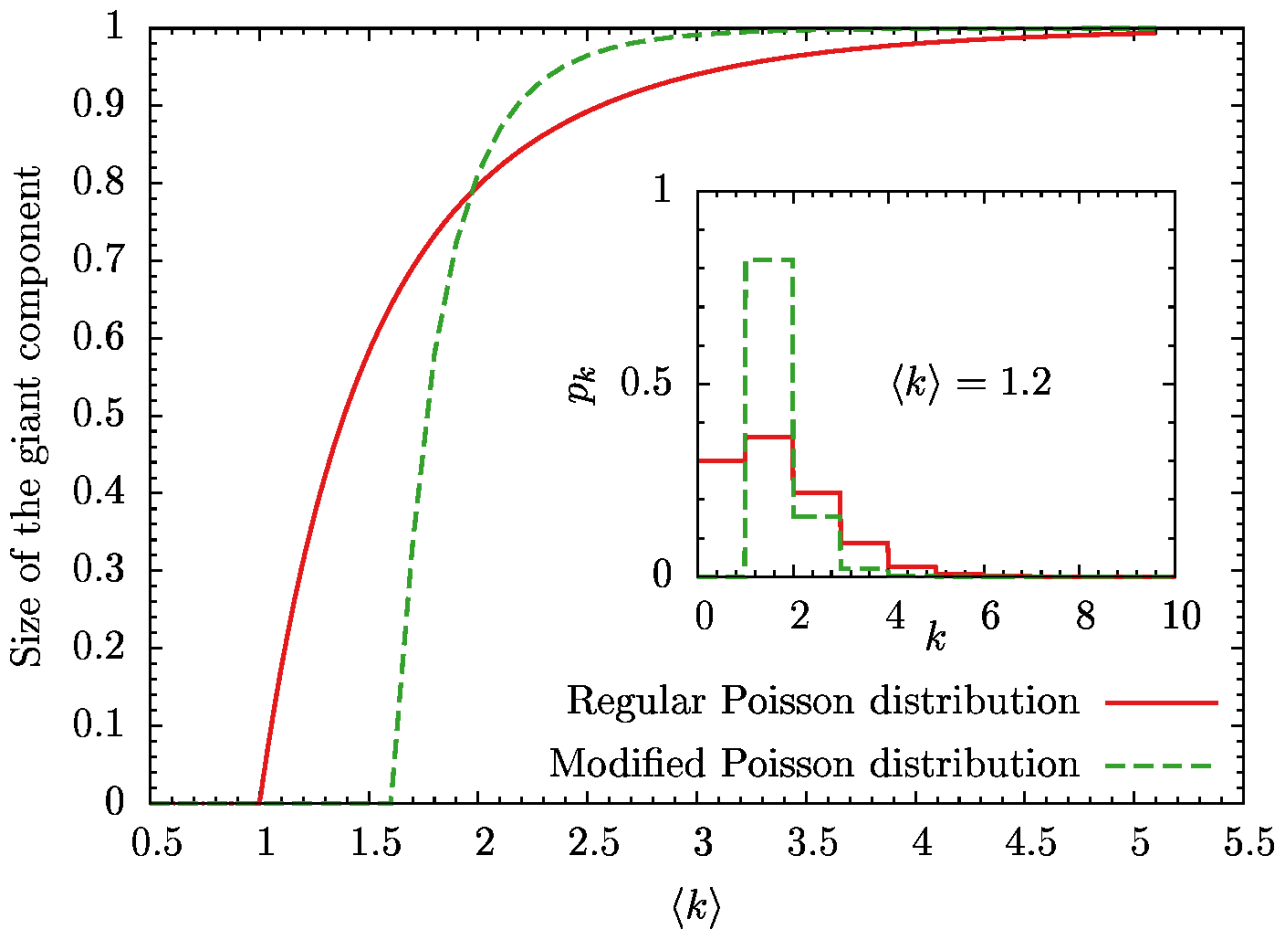
Giant connected component of a Erdős-Rényi random network with a Poisson (dashed green) and modified Poisson (solid red) degree distribution (excluding nodes with 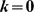) as function the average mean degree of the network. The inset shows the regular and modified Poisson distribution for a mean degree of 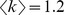 with the same color coding.

The percolation properties of a random network with the modified Poisson degree distribution of Eq. (1) differ slightly from an Erdős-Rényi network, i.e. a random network with a regular Poisson distribution. In the Erdős-Rényi network the percolation transition where a macroscopic connected component emerges as a function of the mean degree occurs at 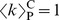. In the case of the modified Poisson distribution, the transition is shifted to 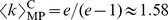, as can be seen in Fig. 1. This is a direct consequence of the fact that no nodes with degree 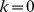 are allowed in the later case. For a modified Poisson network to have low mean degree, 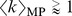, a large fraction of nodes needs to have degree one. In order to achieve this, many of the 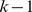 nodes form pairs and are thus isolated from the rest of the network. Additionally, the number of nodes with degree greater than one is strongly reduced compared to the regular Poisson distribution (see inset of Fig. 1). This prohibits the existence of a macroscopic connected component if the mean degree is close to one (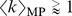). Only when the fraction of nodes with 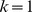 diminishes, a macroscopic giant component can form. In this case, the nonexistence of disconnected 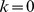 nodes results in larger connected components in general and leads to the stronger increase of the size of the giant components as can be seen in Fig. 1.

Apart from the degree distribution of each block, 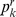, more parameters need to be specified in order to define a realization of a block model ensemble. These are the total number of blocks 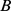, the relative size of each block 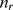, the mean degree of each block 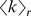, as well as the edges connecting the blocks given by 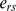. These parameters are, however, not completely independent as the relative sizes 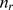 of all blocks must add up to one, 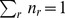, and the sum of all the edges incident to one block is related to its mean degree, 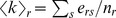. Since we will always consider networks with a given total mean degree 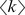, the following constraint will need to be fulfilled, 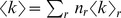.

### 2 Node removal and robustness

Failure in networks is modeled by removing a finite fraction 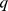 of nodes from the network. We will consider two different strategies for selecting which nodes are removed. The first is *random removal* where the nodes to be removed are selected purely randomly. The second is *targeted removal* where nodes with higher degree are more likely to be removed.

Both types of failures are inspired by real-world technical networks. Random removal is considered to model fatigue of parts or other random influences. Targeted removal is inspired by the fact that highly loaded nodes are more likely to fail or, in the context of critical infrastructure, malicious damage is preferably brought to important nodes.

In the context of block models, where we only model representative nodes in an statistical ensemble, we employ a slight variation of the targeted removal which was also used in Ref. [25]. The targeted criterion is only applied to the selection of blocks where the fraction of nodes to be removed from block 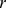 is proportional to 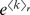 and thus increases with the mean degree of the block. However, within each block no further targeted removal of nodes is performed and nodes are removed at random. In case of all blocks having the same mean degree targeted removal is identical to random node removal.

As a measure of robustness of a network we use the size of the macroscopic component 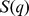 after a finite fraction 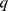 of nodes has been removed. Instead of focusing on the robustness when removing a single fraction 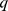, all possible values 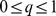 are considered to obtain a sensible measure for the overall robustness of a network. Therefore, we define the robustness as it was proposed in Ref. [11] as
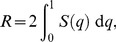
(2)where the factor of 2 serves to adjust the range of 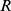 to be 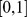. The limiting case 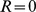 is achieved by networks without a macroscopic component, even when no nodes are removed at all. The opposite limiting case of 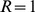 requires a fully connected network where 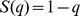.

Following Ref. [25] using the generating function formalism [36] the size of the macroscopic component is calculated using 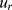, which is the probability that a node in block 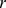 is not connected to the macroscopic component via one of its neighbors. These probabilities for all blocks have to fulfill a system of 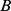 self-consistent coupled equations:
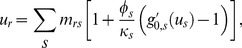
(3)where 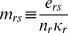 is the fraction of edges in block 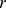 leading to block 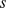, 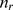 and 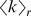 are the relative number of nodes and mean degree of block 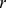, respectively, and 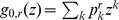 is the generating function of the degree distribution of block 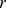 and 
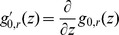
 is its derivative. 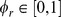 is the fraction of nodes *not* removed from block 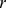. The 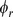 have to be chosen in accordance with the node removal strategy, for example, 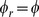 for random removal or 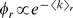 for targeted removal. Since the total fraction of removed nodes is given by 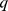, the 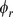 need to satisfy the relation 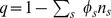. Due to this requirement, the 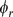 for targeted removal need to be determined by numerically solving 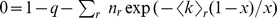 for 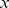 and using the solution 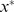 to get 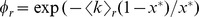.

The solutions of these equations 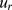 allows for the calculation of the size of the giant connected component 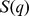,
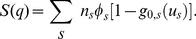
(4)


At this point, a few remarks about the interpretation of the value of 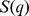 should be made. Since we are parametrizing the system with intensive quantities (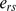, 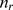, 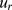, etc.) which specify *fractions* of nodes and edges in infinitely large systems, we cannot differentiate between the existence of single or multiple macroscopic components for a given value of 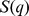. In other words, if two macroscopic components are connected by a single edge (or more generally, any intensive number of edges) the probability of edges between them vanishes in the infinite size limit. Thus, this situation cannot be distinguished from two truly disconnected macroscopic components where no edges exists between the two components. For the purposes of this work, we consider this issue to be unimportant, and we focus on the existence of macroscopic components in the more abstract sense as given by the value of 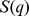 directly.

For each node removal strategy, Eqs. (3) have to be solved for all 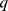 in order to calculate the robustness 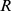 of a specific block model ensemble. In our case, this leads to two different measures of robustness, 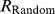 and 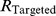, for random and targeted node removal, respectively.

### 3 Evolutionary optimization

In order to consider both robustness measures, 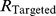 and 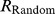, at once, we utilize a multi-objective [27]–[30] evolutionary optimization [37], [38] algorithm. Unlike in the optimization of a single objective, where it is always possible to state if a certain solution 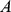 is better, worse or equally good compared to a solution 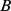, this is not necessarily possible in multi-objective optimization. If a solution 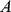 performs better than a different solution 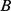 in one objective, but worse in a second objective, no statement is possible which of the solutions is better. Only if solution 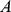 is better than 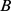 in at least one objective and not worse in any objective it can be considered generally better and it is then said that 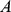 *dominates* 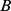. Sets in which no solution dominates any other solution are called *non-dominating*. In general, a multi-objective optimization will not result in a single best solution but in a set of non-dominating solutions which ideally is close to the best possible set of non-dominating solutions, the Pareto-optimal front. These non-dominating sets are very useful to study the trade-off relation between the robustness 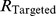 and 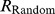 and their relation to the structure of optimal networks.

**Figure 2:**
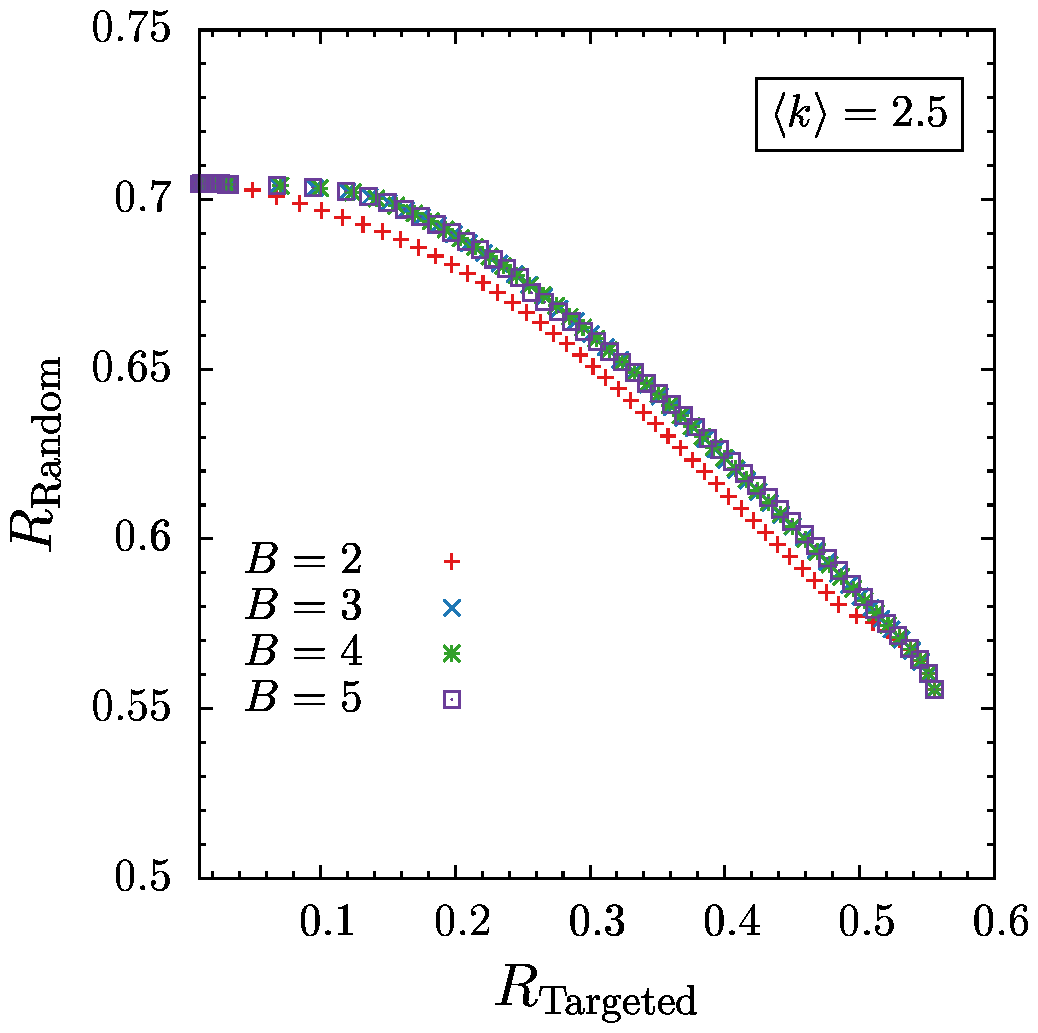
Pareto-optimal fonts of robustness against targeted and random removal of nodes for mean degree 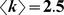 and various number of blocks. For three, four and five blocks the curves match exactly which implies that no more than three blocks are necessary to achieve the best robustness values. Since at the and of the curves all of them match only two or even one block is enough to achieve best robustness.

The algorithm we use here is the so called *S-metric selection evolutionary multi-objective optimization algorithm* (SMS-EMOA) [39]. It is a population based evolutionary stochastic search algorithm which does not utilize any gradient information and is well suited for non-convex and noisy optimization problems. The algorithm does not optimize the objectives directly but instead maximizes the hypervolume in objective-space dominated by the population and bound by a reference point. In the present case of two objectives, the hypervolume is given by the area under the Pareto-curves as, for example, shown in Fig. 2. At each iteration the solution whose removal leads to the lowest decrease in the dominated hypervolume is removed from the population and a new solution is generated by recombination and mutation (for more details see [40]).

Repeating the steps of removing the least contributing solution and generating a new solution not only shifts the solution set closer to the Pareto-optimal front but also leads to a broad distribution along the front, two desired properties of an optimal set of solutions. For completeness, we state the parameters used for the SMS-EMOA: A population size of 50 is used, the crossover probability is 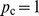, the crossover distribution parameter is 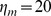, the mutation probability is 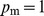, and the mutation distribution parameter is 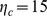.

For each optimization run, we fix the number of blocks 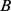 and the mean degree of the complete network, 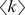. Each block has a modified Poisson distribution as its degree distribution (cf. Eq. (1)), but the average mean degree of each block can vary. Therefore, the free variables subject to optimization, i.e. the search parameters, are the relative size of each block, 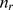, the mean degree of each block, 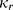, and the entries in the matrix containing edges within and between the blocks, 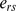. With the sum rules and constraints stated at the end of Section 1, this results in 
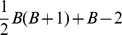
 independent search parameters.

## Results

### 4 Trade-off curves

In Fig. 2 we show robustness values obtained from different optimizations for several numbers of blocks (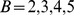), but all with a fixed mean degree of 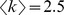. The Pareto-optimal fronts for optimizations with 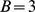, 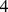, and 5 blocks match exactly. Only the 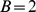 result deviates and yields lower robustness over large parts of the Pareto-optimal front.

The network structures corresponding to the Pareto-optimal solutions for 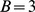, 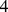, and 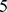 blocks (not shown) are also identical. (Two structures with different 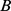 values are considered identical when their structural entropy is the same. See Sec. 5 for more details.) The same behavior was found for other values of the mean degree 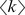, where the results for 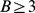 were identical and deviations were only observed for 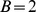.

This leads to the conclusion that three blocks are sufficient to describe networks which are maximally robust against random and targeted node removal. At both extremes of the Pareto-optimal fronts all curves coincide, which means that for optimizing only with respect to one objective (i.e. single-objective optimization), 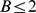 is sufficient to achieve maximal robustness (see Section 5). This is in accordance with the results of Ref. [25], where single-objective optimization was performed, and a 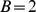 core-periphery and a 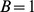 fully random structures were found as optimum for random and targeted node removal, respectively. This is also consistent with the findings of Valente et al. [26] who showed two- and three-peak degree distributions to be optimal when minimizing percolation thresholds of networks subject to random and targeted removal of nodes.

**Figure 3:**
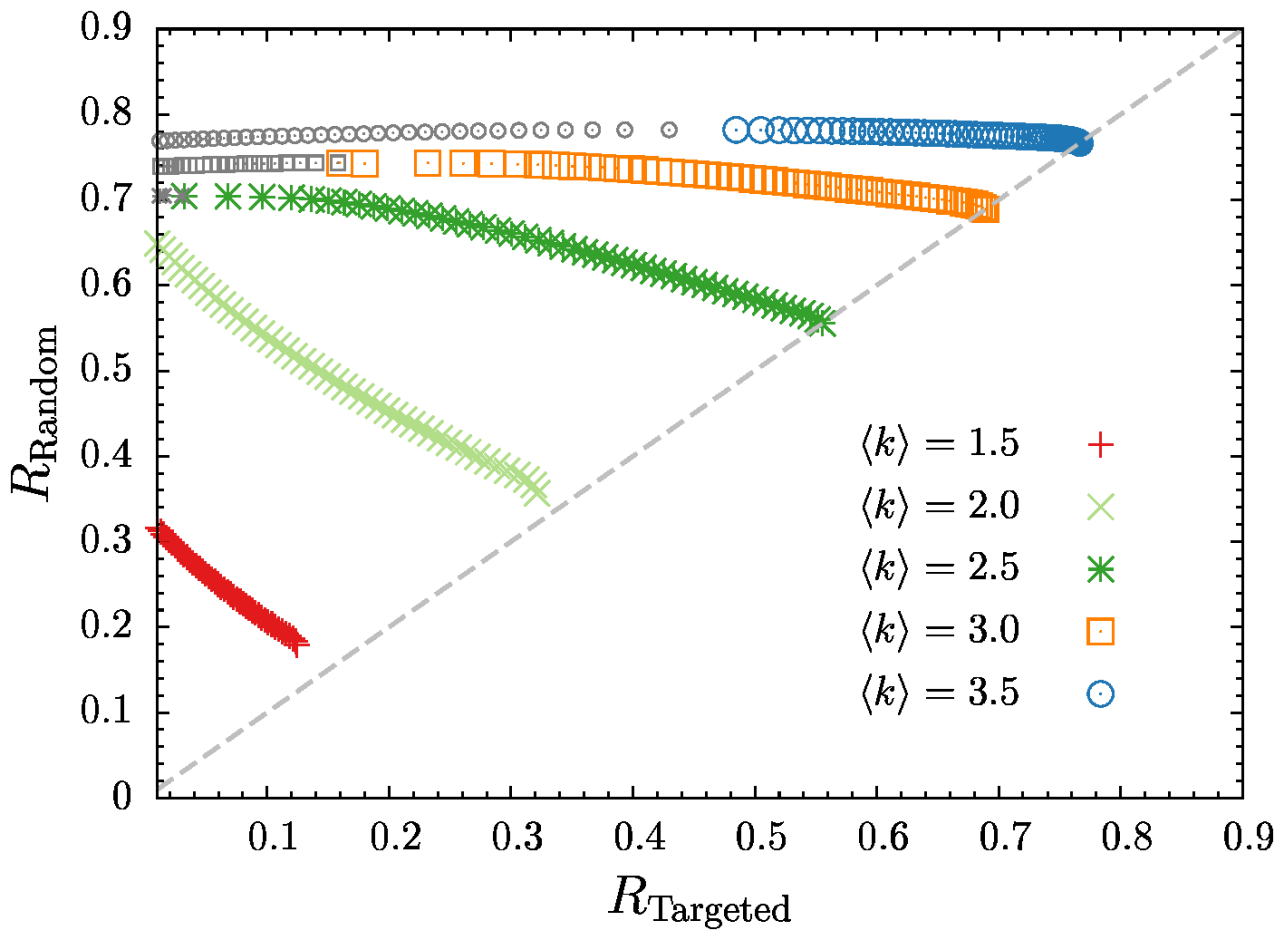
Pareto-optimal fronts of 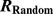 versus 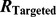 for optimal block model networks with 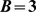 and for various mean degrees (colored symbols). For 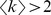, the smaller gray symbols to the left of each Pareto front indicate solutions which maximize 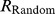 for fixed 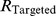 but which are not Pareto-optimal (see main text).

The Pareto-optimal fronts of optimized block model networks with three blocks (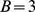) and a mean degree 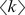 between 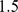 and 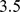 are shown in Fig. 3. As intuitively expected, the general trend where the robustness increases with the mean degree is observed.

For small values of the mean degree, 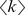
≲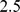, the two types of robustness are in strong trade-off relation: Increasing the robustness against targeted removal strongly decreases the robustness against random removal (and vice-versa).

Pareto-optimal solutions of networks with 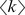
≲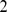 and with the highest robustness against random removal are always found to be most fragile with respect to targeted removal, i.e. at 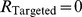. But for 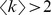, networks with the maximal value of 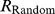 shift to have a finite robustness against targeted removal, 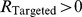. In this case, networks with lower robustness against targeted removal are not accessible via the multi-objective optimization, since they are not Pareto-optimal (i.e. they are dominated by the solutions with maximal 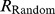, see Refs. [27]–[30]). However, they can be found by performing an optimization with the value of 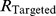 fixed, and such results are shown as the smaller gray symbols in Fig. 3. The Pareto optimal front together with these additional solutions form the whole trade-off curve for each 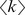.

With increasing mean degree, the trade-off curves become very flat, indicating that a slight sacrifice on the robustness with respect to random removal yields a great enhancement in the robustness against targeted removal. Additionally, the curves increasingly approach the diagonal where 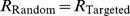, which means that there are solutions which are equally good in both measures.

In general 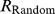 is always greater or equal to 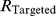, and for 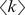
≳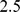, the Pareto-optimal fronts extend to the diagonal. In random networks, nodes with high degree are important for the size of the giant component since they naturally are more likely to connect different components. Due to this, a removal mechanism targeting high degree nodes is able to degrade the giant component easily by removing a relatively small amount of high degree nodes. Therefore, making the degree distribution of a network narrow should increase the robustness against targeted removal since there are less high-degree nodes. In a block model with several blocks a narrow degree distribution implies that all blocks have the same mean degree 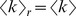. Since, in this work, targeted removal only differentiates between blocks but not between nodes inside the block, targeted and random removal are identical if all blocks have the same mean degree. As a consequence, the robustness values are then equal, 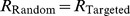.

In contrast, for 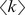
≲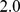, the Pareto-optimal fronts do not extend to the diagonal, which is a consequence of the percolation properties of fully random 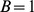 networks (cf. Section 1). For low mean degrees, the giant connected component of a fully random network is very small even without node removal (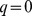). Due to the steep increase of the giant component with increasing mean degree (cf. Fig. 1), it is beneficial to have two blocks with differing mean degree, one higher and the other lower than the total average mean degree 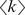. The block having a mean degree greater than 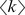, also has a substantial larger giant component, while the giant component of the other block is still small (or even zero). Therefore, the argument presented above for 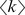
≳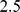, where a finite giant component at 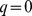 always exists, is not effective for 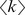
≲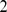. It is always beneficial to have (at least) two blocks in order to have increase the size of the giant component for 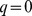.

### 5 Network structures

In our approach, the number of blocks 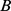 is set a priori and kept fixed during a single optimization procedure. However, networks with different values of 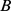 could have equivalent topologies. This can happen if one or more blocks have a vanishing size 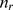 and mean degree 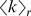, or when two or more blocks can be merged together without altering the ensemble of generated networks.

For a clearer visualization and analysis of block model structures, we reduce the number of blocks by removing insignificant blocks and by merging multiple blocks into one if they are equivalent. For two blocks to be equivalent, we require that the entropy of the merged and the original network ensembles differ by a very small amount. The entropy of the stochastic block model ensemble is simply the logarithm of the total number of networks which can be generated given a specific parametrization, i.e. choices of 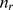 and 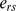. The entropy is a signature of the ensemble, and determines how random it is. If the entropy remains the same after two blocks are merged into one, this means that these two groups correspond simply to an arbitrary subdivision of a larger group, and they do not in fact constrain the topology in any way. The entropy of block model networks is calculated as described in Ref. [41]. We emphasize that, since the topologies in this case are in fact equivalent, the effect of the merging process on the robustness values was found to be negligible.

We now consider the Pareto fronts separately for different values of the mean degree.

#### 5.1 Networks with intermediate mean degree 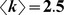


**Figure 4:**
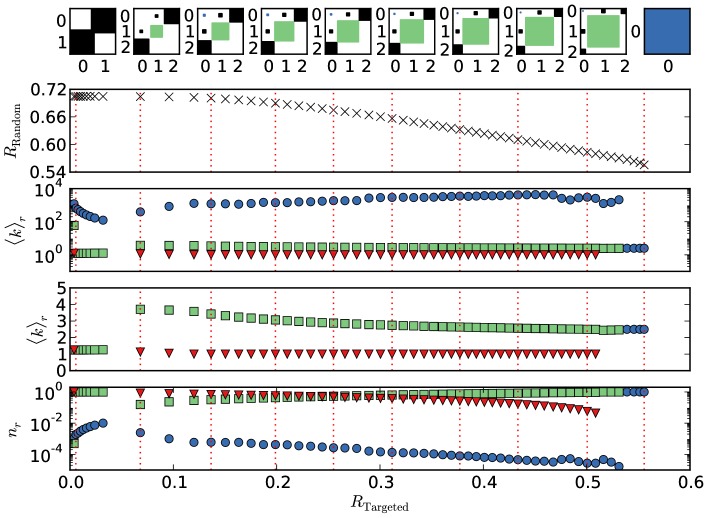
Parameters of the optimized networks as a function of 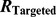 obtained from a three-block optimization with 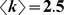. The upper row shows the elements of the edge matrix 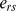, where the areas of the squares is proportional to the logarithm of the element. The positions for which these Hinton plots are shown are marked with dashed lines in the other panels. The second row shows the trade-off curve already displayed in Fig. 2, while the third and fourth display the mean degree of the blocks, on a logarithmic and a linear scale, respectively. The last row shows the relative sizes of the blocks. The coloring of the blocks and their index is determined by their mean degree, where the block with the highest mean degree is shown in blue and always has index 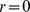, followed by green with 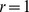 (second highest) and red (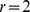, lowest degree).

In Figure 4, the structure and parameters of optimized networks for 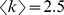 are depicted. The merging procedure is reflected in the fact that the number of blocks indicated by the number indices on the axis and the number of possible squares in the top-row Hinton-plots varies between one and three.

For 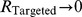 we recover the core-periphery structure found in Ref. [25] where the optimal solution consists of only two blocks. One block is the very large periphery block which contains nearly all the nodes (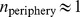) and which has the lowest mean degree possible in this kind of structure 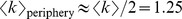. The core block contains only very few (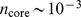) but very high-degree nodes (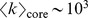). Almost all of the edges are between the core and the periphery.

The core is central for forming the giant component, but takes up only a very small fraction of the network. Therefore, random removal will almost always affect periphery nodes and the giant component will shrink approximately linearly with the number of removed nodes, which is as slow as possible.

On the other hand, the core-periphery structure is maximally fragile with respect to targeted removal, since removing the core completely removes the giant component.

With increasing robustness against targeted removal, a third block emerges for 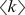
≳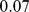 in addition to the core and periphery block. This new block, which we will call the *secluded block* is first of medium size (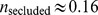) and has a mean degree of 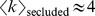. In contrast to the core and the periphery block, it has a substantial amount of edges internally, i.e. edges between nodes within this block (green square in the Hinton plots). The secluded block is only lightly connected to the core block and no edges exist between secluded and periphery block.

Increasing 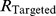 further, the mean degree of the secluded block slightly decreases, while it grows in size. The number of nodes in the periphery continuously decreases and around 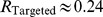 the secluded block is larger than the periphery. For very high 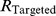
≳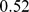 the secluded block dominates and the core periphery structure vanishes. The result is a single block network with a modified Poisson degree distribution, as it was already mentioned in the discussion at the end of Section 4 in connection with Fig. 3.

Considering the complete Pareto-optimal set of solutions, the dominant structure is a three-block structure with a small but very high degree core, a large but low degree periphery and an additional secluded block which has a medium mean degree. Connections only exist between the core and the periphery, and between the core and the secluded block. The structure is best qualified as a modified core-periphery with a regular Erdős-Rényi network attached to the core. The relative size of the secluded Erdős-Rényi block compared to the core-periphery structure grows with an increased robustness against targeted node removal.

#### 5.2 Networks with low mean degree 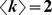


**Figure 5:**
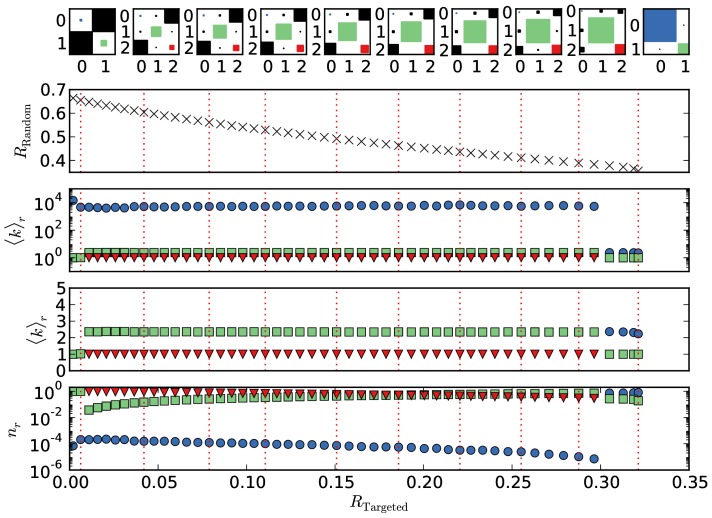
Parameters of the networks along the trade-off curve for the five block optimization with 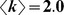. The rows show the elements of the edge matrix 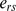, the trade-off curve already displayed in Fig. 2, the mean degree of the blocks on a logarithmic and on a linear scale, as well as the relative sizes of the blocks, from top to bottom, respectively. See caption of Fig. 4 for more details on the coloring and box sizes.

The structures of Pareto-optimal networks with a low mean degree of 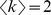 are shown in Figure 5. The resulting structures are overall quite similar to the previously discussed case with 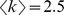. For 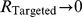 a core-periphery structure results, with an additional secluded Erdős-Rényi block emerging as 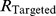 is increased.

However, a striking difference to the situation for 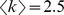 is that the number of edges within the periphery does not vanish but is finite for all structures.

The periphery block always has a mean degree very close to one, 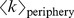
≳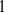, which implies that the majority of nodes has exactly degree one. Therefore, most edges within the periphery produce an isolated pair of two nodes not connected to any macroscopic component (cf. discussion of the modified Poisson distribution in Section 1).

At first glance, this seems contradictory as the giant component is already reduced without any node removal (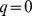). However, this is beneficial for the overall robustness as it allows for the rest of the nodes to have a higher mean degree putting it further above the percolation threshold. As can be seen from Fig. 1, this is especially effective for increasing the macroscopic component of the secluded block as its mean degree of 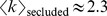 is close to the steep increase in the size of the giant component. This may be viewed simply as an artifact of the specific constraints we have imposed. Perhaps a more realistic scenario would be to impose additionally that the size of the largest component cannot decrease for any value of 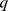 after the optimization. However, this would make the analysis significantly more complicated, and would only affect the outcome of very sparse networks.

Interestingly, this holds for the two-block core-periphery structure as well for 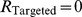. A pure core-periphery structure is especially expected for 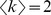, since then the extreme topology of a star can be realized (the corresponding Hinton plot is not shown in Fig. 5). However, a very slight increase in the robustness against targeted removal to 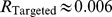, leaves the two-block core-periphery structure intact, but produces a significant amount of pairs in the periphery (see leftmost Hinton plot of Fig. 5). The size of the core jumps from 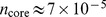 to 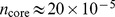 while its mean degree is reduced from 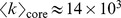 to 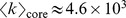. With this structural change, a little robustness against random removal is lost, but the a finite number of edges is realized within the core which provides a finite robustness against targeted removal.

As expected from the discussion at the end of Section 4, the structure with a maximal 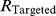 consists of two blocks, one with a mean degree 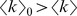 and another with 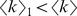. Both blocks in fact form largely independent components since there are very few connections between them. This is a situation similar to the “onion-like” structure found in Ref. [11] when optimizing against targeted node removal while preserving a heterogeneous degree sequence. There, the nodes with higher degree are kept isolated from the rest of the network, hence effectively functioning simply as “bait” for the targeted removal, whereas the rest of the system remains intact.

It is very remarkable that for 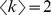 the mean degree of the three blocks stay constant over the complete range of the Pareto-optimal front (apart from the far extremes). The optimal trade-off between the two robustness measures can be achieved by only changing the connection matrix and the relative sizes of the blocks.

#### 5.3 Networks with high mean degree 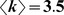


**Figure 6:**
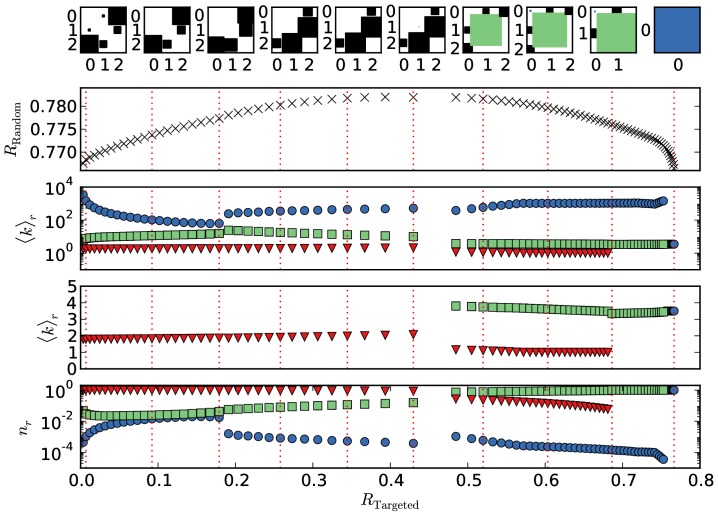
Parameters of the networks along the trade-off curve for the five block optimization with 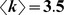. The rows show the elements of the edge matrix 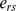, the trade-off curve already displayed in Fig. 2, the mean degree of the blocks on a logarithmic and on a linear scale, as well as the relative sizes of the blocks, from top to bottom, respectively. See caption of Fig. 4 for more details on the coloring and box sizes.

The network structures for a higher mean degree of 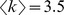 is shown in Fig. 6. The Pareto-optimal part of the front, that is for 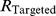
≳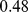, displays the same three-block structures as for 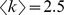 and also reduces to a single block for maximal 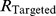 where 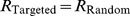 (cf. Fig. 4).

For the part of the trade-off curve to the left of the maximum of 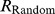 (i.e. for 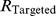
≲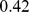), the structures change significantly. A three-block structure prevails but the secluded block ceases to exist. No block has a significant amount of internal edges and all edges connect different blocks. The largest block incorporates most of the nodes, 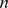
≳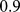, and has the lowest mean degree of 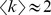. A second block is very small with 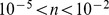 and has a high degree 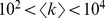 and therefore strongly resembles the core block. The third block is of intermediate size and degree, 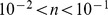 and 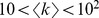, respectively. The mid-sized and the small block are only connected via the largest block since there are no direct edges between them.

For very low robustness against targeted removal, 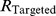
≳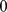, most of the edges are between the core and the largest block with 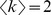. With increasing robustness against targeted removal the number of edges between core and the 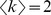 block decreases while more edges emerge between the 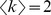 block and the mid-sized block. At around 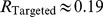 the same number of edges exist from the 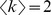 block to both of the other two blocks. For higher 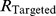 more edges exist between the 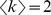 block and the mid-sized block.

This structural evolution can be understood by noting that the largest part of the network always has a mean degree very close to two and acts a connecting layer between the core and the mid-sized block. For low 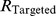, very few edges are between the block with 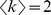 and the mid-sized block, so that a connecting path between two different nodes of the mid-sized block is very likely to traverse one of the few core nodes. Therefore, removing the core quickly fragments the network into small components. On the other hand, increasing the number of edges between the 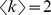 and the mid-sized block, a connecting path between nodes within the mid-sized block is more likely to involve no nodes from the core. The core becomes increasingly unimportant and therefore the robustness with respect to targeted removal increases.

## Discussion

In this paper we investigated the trade-offs between topological robustness of networks against random and targeted node removals. We used the stochastic block model to parametrize arbitrary mixing patterns, and a multi-objective optimization algorithm to obtain the Pareto-optimal fronts. It was found that in order to achieve a Pareto-optimal combination of robustness against random and targeted removal, a network composed of at most three different blocks is sufficient. In many cases the networks along the Pareto-optimal fronts are composed of a hybrid topology, comprised of a core-periphery structure, in addition to a secluded group, which is only sparsely connected to the core of the network, and not at all with the periphery.

At the edges of the Pareto-fronts, where one of the two robustness criteria is maximized, one or two blocks suffice to obtain optimal networks: A two-block structure is maximally robust against random failure, and a fully random network with one block is sufficient in the case of targeted removal. This reproduces the results of Ref. [25], and is also consistent with the earlier findings of Valente et al. [26] who found two- or three-peak degree distributions to be optimal when minimizing percolation thresholds, with networks which are otherwise fully random.

For low mean degrees of the overall network, the optimal robustness values are generally lower than for higher mean degrees and a significant trade-off exists between robustness against random and targeted removal. With increasing mean degree the strong trade-off diminishes and it is increasingly possible to obtain a high robustness with respect to both criteria. This implies that a network optimized against one type of failure does not necessarily lose much of its robustness when it is subsequently optimized against the other type of failure or attack. Hence this shows that increasing the overall connectivity of the network not only has the expected trivial effect of increasing each robustness criterion individually, but to a large extent also allows for them to be fulfilled simultaneously. This suggests that the simple strategy of increasing the total number of edges in the network, if combined with the optimal large-scale structures present in the Pareto-optimal front, can be much more beneficial than could be expected otherwise.

A comparison of the large-scale structures we find with the ones observed in empirical systems [31], [42], [43] is a natural and important extension of this work, and one we intend to pursue in the future. The most appropriate approach is to search for precisely the same type of model we are using in the analysis, which can be done by inferring the parameters of the stochastic block model itself from empirical data, which is a very active field of research [32], [33], [44], [45]. In fact, core-periphery structures have already been detected, such as the topology of the Internet at the autonomous systems level presented recently in [46]. However, to our knowledge, an empirical verification of the specific structures we have found has not yet been made.

In this work we have considered maximally robust networks that are obtained when very few constraints are imposed. This gives us fundamental limits on the multi-objective optimization against random failures and targeted attacks. However, in empirical systems, exogenous constraints are almost always present, such as geographical confinement, and restrictions due to functional performance. In previous studies [11], [47], the optimization against targeted node removal was considered when the degree sequence of an empirical network is preserved. It was found that an assortative structure emerges as the optimum in this case, where nodes become connected with other nodes with similar degree. This has been obtained as well by imposing similar constraints with the block model approach in Ref [25]. However, it is still unknown how the multi-objective optimization would behave when these constraints (and other more realistically motivated ones) are simultaneously imposed. We leave these questions for future work.

## Supporting Information

Dataset S1 Compressed file containing the final results of all optimizations shown here. The name of each file contains the respective number of blocks and the mean degree of the network. The columns inside each file are aranged in the following order: both robustness values, mean degree of each block, relative sizes of the blocks, elements of the connection matrix.
(7Z) Click here for additional data file.
